# Diabetes Detection and Prevention in Dermatology

**DOI:** 10.5826/dpc.1104a131

**Published:** 2021-10-01

**Authors:** Alexandra Ngo, Luise Froessl, John Wesley McWhorter, William Brett Perkison, Rajani Katta

**Affiliations:** 1 Department of Psychiatry, Baylor College of Medicine; 2 Baylor College of Medicine; 3 Culinary Nutrition of the Nourish Program, at the Michael & Susan Dell Center for Healthy Living at The University of Texas School of Public Health; 4 Department of Epidemiology, Human Genetics, and Environmental Science at the at the University of Texas School of Public Health in Houston; 5 McGovern Medical School at the University of Texas Health Science Center at Houston

**Keywords:** diabetes prevention, diabetes in dermatology, diabetes skin findings, psoriasis and diabetes, diabetes screening

## Abstract

We are currently in the midst of an international epidemic of diabetes mellitus (DM) and prediabetes. The prevalence of DM in the United States is estimated at 9.4% of the population across all ages, while an estimated 1 in 3 Americans (33.9%) has prediabetes. According to the WHO, about 60 million people suffer from diabetes in the European Region. Dermatologists may play an important role in tackling this epidemic via efforts to improve early detection of both diabetes and prediabetes. Dermatologists often treat patients with, or at risk of, diabetes. This includes patients who present with cutaneous manifestations such as acanthosis nigricans, as well as patient populations at increased risk, including those with psoriasis, hidradenitis suppurativa, and polycystic ovarian syndrome. Simple screening guidelines can be used to identify patients at risk, and screening can be performed via a single non-fasting blood test. The diagnosis of prediabetes is a key feature in diabetes prevention, as interventions in this group can markedly reduce progression towards diabetes. In addition to referral to a primary care physician, dermatologists may refer these patients directly to structured behavioral lifestyle intervention programs known as diabetes prevention programs. A significant portion of the population lacks routine care by a primary care physician, and current data indicates need for improvement in diabetes screening and prevention among patient groups such as those with psoriasis. These factors highlight the importance of the dermatologist’s role in the detection and prevention of diabetes.

## Introduction

We are currently facing an international epidemic of diabetes mellitus and prediabetes. The prevalence of DM in the United States is estimated at 9.4% of the population across all ages, while an estimated 1 in 3 Americans (33.9%) has prediabetes [1]. According to the World Health Organization, about 60 million people suffer from diabetes in the European Region [2].

Dermatologists may play an important role in fighting against this epidemic. Efforts to identify and screen patients at risk can improve early detection of DM, and help identify those with prediabetes. The identification and treatment of patients with prediabetes is considered a key aspect in diabetes prevention.

There are 3 main factors making the dermatologist’s potential impact on DM epidemic further possible and more important than ever. First, dermatologists often treat patients with, or at risk of, DM. This includes patients who present with cutaneous manifestations of DM, such as acanthosis nigricans, as well as patient populations at increased risk for DM, including those with psoriasis, hidradenitis suppurativa, and polycystic ovarian syndrome [3–6]. Second, dermatologists can use simple screening guidelines to identify patients at risk for DM [7]. Patients at risk may then be screened via a single non-fasting blood test. Third, diagnosing prediabetes is a key feature in DM prevention, as interventions have been shown to markedly reduce progression to DM. In addition to referral to a primary care physician (PCP), dermatologists may refer patients with prediabetes to diabetes prevention programs, behavioral lifestyle intervention programs that are cost-effective and effective [8].

Although DM screening and prevention might be typically performed by the PCP, data indicates that a significant portion of the population lacks routine care by a PCP. According to a recent study, only 75% of American adults reported a source of primary care in 2015 [9]. Some subgroups saw an even more marked decline in coverage, specifically younger patients, less medically complex patients, and those belonging to minorities. In parallel, the prevalence of type 2 DM has been steadily increasing, particularly among adolescents and young adults. Age of onset before 40 years has been associated with more severe long-term outcomes [10], emphasizing the importance of early diagnosis and continuous management.

A significant percentage of dermatology patients at increased risk for comorbidities lack care by a PCP. This draws attention to the potential role that can be played by dermatologists in diabetes detection. A recent study found that among patients suffering from psoriasis, 21.6% of men and 16.9% of women received no primary care visits within a year from their dermatologist visit. In hidradenitis suppurativa, 28.1% of male patients and 22% of female patients were not visited further [11].

Furthermore, current data indicates that there is space for improvement in DM screening and prevention. Although multiple large-scale studies have found that patients with psoriasis are at higher risk for DM and other systemic comorbidities [4, 12], data indicates that healthcare providers are not adequately screening psoriasis patients for metabolic risk factors [13]. Dermatologists rarely screen their patients for risk factors such as glucose levels (1.2%), BMI (9.7%), or blood pressure (2.6%) [13]. Even among PCPs and cardiologists, survey studies reveal that many do not routinely screen patients with psoriasis for cardiovascular risk factors [14].

### The Importance of Early Detection

Dermatologists may interact with patients with DM at all stages of the disease, including those who are not yet diagnosed. Despite the availability of simple screening measures, DM often goes undiagnosed for years. Much of this is because patients may be asymptomatic for years. These years, however, are critical. While patients are at high risk for the development of micro- and macrovascular complications, these have not yet become irreversible.

Early detection of DM and institution of early treatment may profoundly impact the course of the disease [15]. DM impacts multiple organ systems, including the brain, heart, kidneys, and skin, with significant impacts on morbidity and mortality. The National Diabetes Statistics Report found DM to be the 7^th^ leading cause of death in the United States in 2015 [1].

### Skin Findings Strongly Associated with Insulin Resistance and DM

Approximately 85 million Americans (1 in 4 individuals) were evaluated by a physician for a skin disease in 2013 [16]. Certain cutaneous findings should trigger a high level of suspicion and screening for DM. In a prospective observational study conducted in a diabetes clinic, cutaneous lesions were seen in over 98% of those with type 2 DM and 34% of those with Type 1 DM [17]. While there was a higher prevalence (98%) of skin manifestations in patients with DM for more than 5 years, cutaneous manifestations were still present in 80% of patients with DM for less than 5 years [17].

Acanthosis nigricans (AN) is a classic cutaneous manifestation of DM that is associated with hyperinsulinemia. Studies indicate that elevated levels of insulin and insulin-like growth factor may act to increase epidermal keratinocyte and dermal fibroblast proliferation, resulting in hyperpigmented, thickened, and velvety areas of skin [18].

Cutaneous infections are particularly prevalent in those with DM. In a prospective study of 750 patients, 79% had skin manifestations, with the most common (in 47.5% of patients) being cutaneous infections, including bacterial, viral and fungal [19].

Diabetic dermopathy (DD) warrants prompt evaluation for DM. The atrophic, hyperpigmented macules of DD are classically seen on the shins, and are considered a common cutaneous manifestation of DM [20]. Notably, the condition is also significantly associated with microangiopathic complications of DM such as retinopathy [20] and cardiovascular disease.

Necrobiosis lipoidica is overall rare; although estimates vary, it is believed to impact about 1% or less of patients with DM [21]. Its presence should however lead to consideration of DM, as underlying DM has been seen in anywhere from 11% [22] to 65% [23]. It may be seen prior to the development of DM as well, and reviews estimate that up to 14% of patients may later develop DM [21].

### Nonspecific Dermatologic Findings with Higher Prevalence in Patients With DM

It is important to note that some dermatologic conditions that are commonly seen in the general population have an even higher prevalence among those with DM. Cutaneous xerosis is a common skin complaint overall. Multiple studies examining the frequency of skin manifestations in DM have showed a high prevalence of xerosis in both type 1 and type 2 DM [24]. As xerosis often appears on the lower extremities, it is also an important element in the prevention of foot complications. One study indicated that xerosis of the feet in those with DM was associated with 3 times the number of superficial fissures [25].

Similarly, acrochordons have a prevalence of about 25% in the adult population [3] and a correlation with abnormalities in carbohydrate metabolism has been established [26].

### Skin Diseases Associated with an Increased Risk of DM

In addition to the dermatologic manifestations of DM, physicians must also be familiar with dermatologic diseases that are associated with a higher risk of DM. These include psoriasis, hidradenitis suppurativa, and polycystic ovarian syndrome.

Research indicates a significantly increased risk for DM and other metabolic abnormalities in patients with psoriasis. A meta-analysis of 27 observational studies found that psoriasis is associated with both an increased prevalence and incidence of DM [4]. A 13-year study of over 52,000 patients with psoriasis in a nationwide Danish cohort, reported a significantly increased risk of new-onset DM [12]. Importantly, this risk was significantly higher for those with severe skin disease as compared to those with mild disease. This increased risk is attributed to a combination of the systemic inflammation seen in those with psoriasis and the higher prevalence of obesity [12, 27]. A relationship between pro-inflammatory cytokines in psoriasis and systemic effects has been revealed, suggesting their contribution to insulin resistance, weight gain, and cardiovascular events [27]. In addition, the psychosocial impact of psoriasis may contribute to behavioral risk factors for the development of obesity and DM [28].

Recent research indicates a higher risk of DM in patients with hidradenitis suppurativa (HS) as well. A systematic review and meta-analysis conducted by Phan et al [29] identified a small but statistically significant association of DM with HS. A recent cross-sectional study by Ahmad et al [30] suggests benefits of screening for DM, as even subgroups of HS patients without traditional risk factors for DM (including age and high BMI) were found to have a high incidence of abnormal screening results. Although additional research is needed, dermatologists should be aware of this association.

It is important for dermatologists to diagnose polycystic ovarian syndrome (PCOS), given that these patients have a higher risk of DM and often present to the dermatologist with commonly associated dermatologic manifestations, such as hirsutism, acne, acanthosis nigricans, alopecia or seborrhea [31]. A 2020 longitudinal study of PCOS patients assessed the metabolic changes in patients with PCOS and found a high prevalence of adverse changes in glucose metabolism, with deterioration of beta-cell function. The authors emphasize the importance of early detection and intervention in this group [32].

One prospective cohort study found a DM prevalence of close to 40% in patients with PCOS, as compared to 6% in the general population of a similar age [6]. Patients who are normal weight are at higher risk as well: one long-term longitudinal study found a 3-fold increase in risk of developing DM in normal-weight PCOS patients compared with normal-weight women without PCOS [33].

### The Dermatologist’s Role in DM Screening and Prevention

Dermatologists can expect to see patients with prediabetes and DM, some of them undiagnosed. In fact, of the approximately 1/3 of the US population with prediabetes, 90% are unaware of their status [1]. Risk increases with age, and DM screening is recommended for all patients over the age of 45.

Dermatologists may take a more active role in DM screening and prevention via three main routes. First, by identifying patients at risk for DM. Second, by ordering screening labs. And finally, by referring to PCPs and diabetes prevention programs when indicated.

### Identifying Patients at Risk for DM

Identifying patients at risk for DM is the first step in diabetes prevention and represents an important opportunity for dermatologists. Many dermatology patients, especially those who are younger, may not have a primary care physician [34]. For some, a dermatologist may be their only regular contact with the healthcare system, as with patients who are seen for annual skin cancer screening exams or those who are treated for acne.

Recommendations for DM screening differ based on risk factor profile. Table 1 summarizes screening recommendations by the American Diabetes Association.

In terms of patients with skin disease, guidelines are not yet defined, and this area needs further research and consensus. Certainly, many patients diagnosed with acanthosis nigricans, or recurrent cutaneous fungal infections would benefit from screening.

Psoriasis patients who are overweight or obese should also be considered for screening. Even in the absence of a high BMI, however, those with severe psoriasis may require screening. Studies have demonstrated increased risk of DM even in the absence of traditional risk factors, and severity of skin disease has been linked to a higher risk for metabolic comorbidities [12, 36]. Patients with skin disease severe enough to warrant biologic therapy should therefore also be screened for DM.

### Screening Laboratory Tests

Screening may be accomplished by several methods, including via measurement of hemoglobin A1C level. This is a single non-fasting blood test, and can be used to diagnose prediabetes or DM.

Diabetes mellitus (DM) is classified into two main categories: Type I and Type II. In the last 50 years Type II DM has steadily increased in prevalence and now accounts for 90–95% of cases in the US [35]. According to the American Diabetes Association (ADA), diagnostic criteria for type II diabetes includes a Hemoglobin A1C level of 6.5% or above, a fasting blood glucose test of 126 mg/dL or above, an impaired glucose tolerance test, and/or a random blood glucose test of 200 mg/dL or above [7].

### Prediabetes Diagnosis

While early detection of DM remains a high priority, an increased focus has been paid to prediabetes as well. Identifying these individuals is a key aspect of diabetes prevention, as evidence-based interventions have shown a relative risk reduction of 40–70% in adults with prediabetes [37].

The ADA defines prediabetes as a hemoglobin A1C value of 5.7% to 6.4% or a fasting plasma glucose of 100–125 mg/dL [7], while the WHO defines prediabetes as a fasting plasma glucose of 110 – 125 mg/dL or an impaired glucose tolerance test.

Prediabetes is considered an intermediate state of hyperglycemia that has not quite surpassed the DM threshold. It has been shown that conversion from prediabetes to DM occurs at a rate of up to 19% annually, with the likelihood of progression increasing with time [37,38]. Importantly, prediabetes may also revert to normoglycemia. In a Cochrane systematic review, 47 of 103 prospective cohort studies indicated that regression back to normoglycemia was as high as 59% within 5 years and 42% within 6–11 years [38].

Treatment of prediabetes is crucial because individuals have a window of time where lifestyle changes or medication may be successful in preventing the development of DM. The role of lifestyle change in particular has received much attention. Although the number of drugs available for DM treatment has quadrupled since 1995, there has only been an estimated 8% improvement in glycemic control nationwide. This has led to a renewed emphasis on the importance of lifestyle interventions, which can significantly reduce the risk of DM complications [39].

### Interventions

For patients diagnosed with prediabetes or DM, referral to primary care is required. In addition, dermatologists may directly refer patients with prediabetes to structured lifestyle intervention programs known as certified diabetes prevention programs (DPPs). These programs significantly reduce the risk of progression to DM. They also empower patients, an underappreciated yet significant benefit [40].

A randomized placebo-controlled trial documented that an individualized, structured lifestyle intervention program was able to reduce progression to DM by 58% [41]. This data led to the development of diabetes prevention programs.

In the United States, the CDC has designed a diabetes prevention program based on this evidence that has been reformatted for small group sessions. Similarly, in Europe, the IMAGE project (Development and Implementation of a European Guideline and Training Standards for Diabetes Prevention) has defined standards for quality of diabetes prevention programs in the EU [42]. DPPs last 1 year, and focus on patient education and techniques that encourage behavioral change. Patients learn techniques to help encourage healthy eating, incorporate physical activity, and use healthy coping strategies. To help build and maintain the morale, trained lifestyle coaches are scheduled to meet with patients in group settings a total of 22–24 times throughout the program [8].

The CDC maintains a database of certified programs, which are administered through a variety of healthcare providers, national and local non-profits, and telehealth programs. The CDC provides an online search tool to check for availability of programs in the local area. In the US, coverage is provided for Medicare patients who meet the medical qualifications. Some commercial insurance plans and employers may also provide coverage for the program cost. For those without such coverage, program costs will vary; according to the American Medical Association, costs to the patient for the entire 1-year program in the US were estimated at $400–$500 total [43].

In the EU, DPPs are adapted and implemented at a national level, such as, for example, in Germany, one of the leading countries in diabetes prevention [44]. A Europe-wide multi-center study showed a significant increase in health-related quality of life indicators in patients across Europe, with both diabetes and prediabetes, who participated in DPPs [39].

### The Benefits of Diabetes Prevention

It is well-known that DM results in serious health impacts, including increased morbidity, cardiovascular complications, and increased healthcare costs [35]. For an individual patient, reducing the risk of this chronic disease may be life changing.

For those who enroll in a DPP, weight loss is another benefit. To achieve certification, a DPP must demonstrate weight loss of at least 5% in a certain percentage of participants [43].

In patients with psoriasis, weight loss is an important benefit. The medical board of the National Psoriasis Foundation in 2018 published their dietary recommendations for those with psoriasis. The authors “strongly recommend” weight reduction in those who are overweight or obese [45]. Weight loss may be considered adjunct therapy in psoriasis [46–48] as it may result in improvement of skin disease, with studies demonstrating an improvement in PASI scores. Weight loss may also improve response to systemic psoriasis therapies [49].

## Conclusion

Dermatologists may play an important role in combating the current DM epidemic. Early detection of DM and treatment initiation may significantly improve patient outcomes and reduce the risk of serious complications. Using demographic factors and simple screening tools in the office, patients at risk for DM may be easily identified. Patients at risk may be screened via a single non-fasting blood test or referred to their PCP for further evaluation.

Efforts to identify those with prediabetes is a key aspect of diabetes prevention, as lifestyle intervention programs are now accessible for many and demonstrate impressive results in reducing the risk of progression to DM. These programs also set weight loss goals, which may have additional skin disease benefits. For patients with skin diseases such as psoriasis, these measures will improve both overall health and skin health.

## Figures and Tables

**Table 1:** Summary of screening recommendations by the American Diabetes Association (ADA) [35].

All patients 45 and over should be screened every 3 years	
Asymptomatic patients under the age of 45 with any of the following 8 risk factors AND a BMI ≥ 25 kg/m^2^ should undergo screening (If Asian American, then BMI ≥ 23 kg/m^2^)	
Demographics	High-risk race/ethnicity (Black, Hispanic/Latino, American Indian, Asian American, or Pacific Islander)
Family and Social History	Physical inactivity First-degree relative with diabetes
Medical History	Hypertensive (≥ 140/ 90 mmHg or on therapy for hypertension) Other clinical conditions associated with insulin resistance such as severe obesity, acanthosis nigricans History of cardiovascular disease Polycystic ovary syndrome (PCOS)
Laboratory Values	HDL – C < 35 mg/dL and/or triglycerides >250 mg/dL
Follow-up recommendations: If results are normal, repeated screening should occur at least every 3 years Women who were diagnosed with gestational diabetes should undergo screening every 3 years Patients with prediabetes should undergo screening annually	
